# Serologic Evidence of *Lyssavirus* Infections among Bats, the Philippines

**DOI:** 10.3201/eid0803.010330

**Published:** 2002-03

**Authors:** Paul M. Arguin, Kristy Murray-Lillibridge, Mary E.G. Miranda, Jean S. Smith, Alan B. Calaor, Charles E. Rupprecht

**Affiliations:** * Centers for Disease Control and Prevention, Atlanta, Georgia, USA; † Research Institute for Tropical Medicine, the Philippines;

**Keywords:** rabies, *Lyssavirus*, Chiroptera, Philippines

## Abstract

Active surveillance for lyssaviruses was conducted among populations of bats in the Philippines. The presence of past or current *Lyssavirus* infection was determined by use of direct fluorescent antibody assays on bat brains and virus neutralization assays on bat sera. Although no bats were found to have active infection with a *Lyssavirus,* 22 had evidence of neutralizing antibody against the *Australian bat lyssavirus* (ABLV). Seropositivity was statistically associated with one species of bat, *Miniopterus schreibersi*. Results from the virus neutralization assays are consistent with the presence in the Philippines of a naturally occurring *Lyssavirus* related to ABLV.

During the past decade, bats have been associated with a number of newly recognized zoonotic agents, including Hendra, Menangle, Nipah, and Ebola viruses and the *Australian bat lyssavirus* (ABLV) (*1*–*5*). ABLV and classic *Rabies virus* (RABV) are members of the genus *Lyssavirus.* These viruses are genetically similar and cause indistinguishable clinical syndromes in infected mammals. In the United States, where endemic canine rabies has been eliminated through vaccination and animal control, bat-associated variants of RABV have accounted for 24 (75%) of the 32 cases of human rabies reported since 1990 (*6*,*7*). Of the nearly 30,000 laboratory-confirmed cases of animal rabies reported worldwide in 1997, 4% were in bats (*8*). However, not all countries are included in this survey, and surveillance methods vary between countries included in the compilation. Bat-associated rabies cases in humans are likely underreported in this global surveillance report because not all countries report a history of animal exposure or type the virus variants.

In the Philippines, where approximately 350 cases of human rabies are diagnosed clinically each year, attribution of the animal associated with the exposure is based on history (*8*). Previous surveys for rabies in Philippine bats conducted in the 1950s and 1960s failed to document active rabies infection in the animals examined (*9*,*10*). The increasingly recognized role of bats in the global maintenance and transmission of viral infections, the recent discovery of rabies among bats in Australia, and the unknown proportion of rabies cases in Southeast Asia potentially attributable to bats prompted this initiation of active surveillance for lyssaviruses in Philippine bat populations.

## Methods

### Collection of Specimens

From June 25 through September 11, 1998, bats were nonrandomly collected from multiple sites on six different islands in the Philippines (Figure). Sites were chosen on the basis of local reports of known bat colonies or after investigation of likely habitats, such as caves, church belfries, or orchards (*11*,*12*). Insectivorous and small fruit bats were captured during the day in fine-mesh, long-handled butterfly nets and at night in mist nets. Larger fruit bats were also obtained from hunters. Thick leather gloves were worn when captured bats were transferred into individual muslin pouches for transportation.

**Figure:**
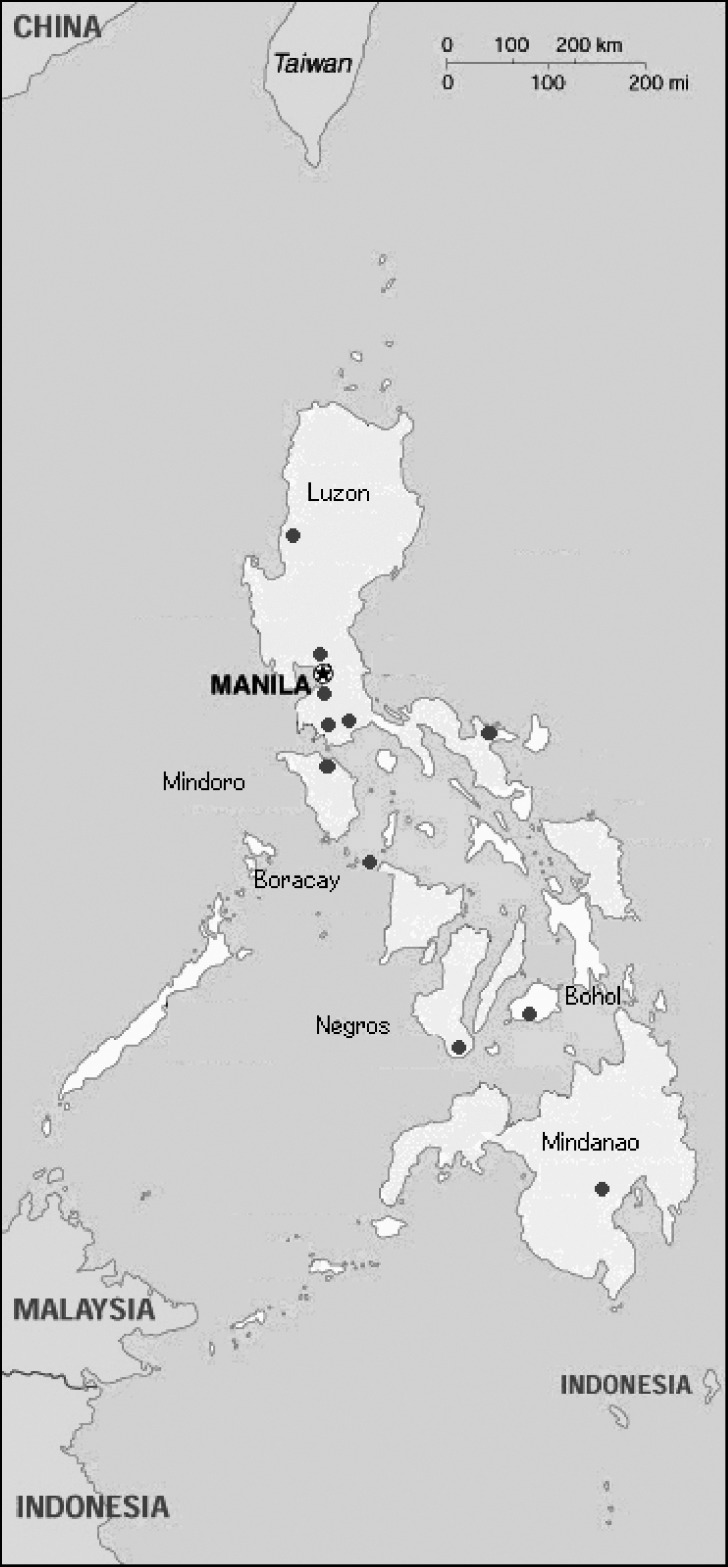
Collection sites for bats used in active surveillance of lyssaviruses in the Philippines.

Bats were anesthetized by a 0.05- to 0.1-mg intramuscular injection of ketamine hydrochloride and euthanized by intracardiac exsanguination. All blood was transferred from the collecting syringe into serum separator tubes and refrigerated until centrifugation. Serum was decanted into individual screw-topped vials and frozen at –20°C. Bats were identified to species by using a key based on gross morphology (*13*). The brains of all bats were removed surgically and frozen in individual containers. Additional organs (e.g., liver, spleen, and lungs) were also harvested from each bat and stored either in a freezer at -70°C or in 20% formalin for future studies. Carcasses of representative specimens were stored in formalin for archival purposes.

### Direct Fluorescent Antibody (DFA) Testing of Brains

At the Research Institute for Tropical Medicine in Manila, the bat brains were thawed and multiple impressions were prepared for DFA testing (*14*). Microscope slides were fixed in cold acetone and allowed to dry. Brain impressions were stained with fluorescein isothiocyanate (FITC)-conjugated anti-rabies monoclonal antibodies (Fujirebio Diagnostics, Malvern, PA) and examined under a fluorescent microscope for *Lyssavirus* antigens. This monoclonal antibody preparation reliably detects infection with all known lyssaviruses, including both classic RABV and ABLV (*5*,*15*–*17*).

### Serologic Testing for Neutralizing Antibodies

At the Centers for Disease Control and Prevention (CDC), the presence of virus-neutralizing antibodies was determined by a modification of the rapid fluorescent focus inhibition test (RFFIT) (*18*). Two different challenge viruses were used: the routine rabies challenge virus standard (CVS-11) and an isolate of ABLV adapted to cell culture. Viruses and bat serum samples were diluted with Eagle minimum essential medium containing 10% fetal calf serum (EMEM10) and antibiotics to reduce bacterial and fungal contamination as described (*18*). EMEM10 was also used in the growth of murine neuroblastoma (MNA) cells, which were used to propagate and grow sufficient quantities of each virus, and in the modified RFFIT assays. CVS-11 was obtained from stocks at CDC. The ABLV isolate (*Saccolaimus flaviventris* [sm4068]; Australian Animal Health laboratory, Geelong, Australia) was originally obtained from an insectivorous bat in Australia and was amplified by passage in BHK and MNA cells at CDC.

All bat serum samples were thawed and placed in a 56°C water bath for 30 minutes to inactivate complement. Serum samples were then diluted to 1:10 if possible. Samples with insufficient volume were screened at a higher dilution. The RFFIT was conducted by using Lab-Tek 8-well glass slides with covers (Nalge Nunc International, Naperville, IL). Sera were screened for antibody by incubating 100 μL of diluted serum with 100 μL of ABLV or CVS-11 that had been diluted to contain approximately 100 infectious units when incubated for 90 minutes at 37°C in a CO_2_ incubator. MNA cells (approximately 75,000 cells/200 μL) were added to each serum-virus mixture, and the incubation was continued. After 48 hours, culture medium was removed, and the slides were fixed in acetone, air-dried, and stained for residual virus with FITC-conjugated anti-rabies monoclonal antibodies. A sample was defined as positive for neutralizing antibody if at least a 90% reduction in infectious centers as observed relative to the positive control. All positive samples were retested at increasing dilutions to estimate endpoint antibody titers. Standard human rabies immune globulin (HRIG) diluted to contain 2 IU/mL antibodies was used as a positive serum control for all tests. The titer of HRIG ranged from 1:125 to 1:625 against both ABLV and CVS-11.

Results from the serologic testing were used to detect patterns in seropositivity by location or type of bat, by using the Chi-square test.

## Results

### Collection of Specimens

Of the 821 bats collected, all but three were identified to species (Table 1). The collection resulted in 14 different species of both insectivorous and frugivorous bats representing five of the six families of Chiroptera believed to be present in the Philippines. Fifty-one percent of the bats were female, including 22 that were pregnant and 6 that were suckling infants. All bats appeared to be healthy except one with an enlarged spleen and three that appeared to have a mange-like condition. Since some bats died during collection and processing, serum could not be collected from all the bats.

**Table 1:** All bats caught on six islands in the Philippines and tested for *Rabies virus* antigen by direct fluorescent-antibody assay, June 25 to September 11, 1998

Bat species	Island of origin					
	Luzon	Bohol	Boracay	Mindanao	Mindoro	Negros
*Saccolaimus saccolaimus*		53				54
*Taphozous melanopogan*	96					
*Megaderma spasma*	16					
*Hipposideros diadema*		16	1	2		
*Rhinolophus* spp*.*	6			3		
*Mineopterus schreibersi*				14		
*Philetor brachypterus*				24		
*Scotophilus kuhlii*	105			1		95
*Cynopterus brachyotis*	3			12	1	4
*Eonycteris spelaea*	1		6	2	1	
*Macroglossus minimus*	4			3	3	
*Ptenochirus jagori*	36			4	6	
*Pteropus hypomelanus*			27			
*Rousettus amplexicaudatus*	1	112	98	6	1	1

### DFA Testing of Brains

Brains from all 821 bats were tested for the presence of RABV antigen by DFA. None of the bats had detectable antigen consistent with an active infection with rabies or a related *Lyssavirus*.

### Serologic Testing for Neutralizing Antibodies

Of the 821 bats collected, 231 had sufficient volume and quality of serum to be diluted to 1:10 and successfully screened at 1:20, after being combined with the challenge virus (Table 2). An additional 43 specimens were screened at higher dilutions. Remaining samples contained insufficient volumes or could not be tested because of hemolysis.

**Table 2:** All bats caught on five islands in the Philippines and screened for neutralizing antibodies against *Australian bat lyssavirus* at a 1:10 serum dilution

Bat species	Island of origin				
	Luzon	Bohol	Boracay	Mindanao	Mindoro
*Saccolaimus saccolaimus*		23			
*Taphozous melanopogan*	30				
*Megaderma spasma*	4				
*Hipposideros diadema*		4	1	2	
*Rhinolophus* spp.				2	
*Mineopterus schreibersi*				11	
*Philetor brachypterus*				13	
*Scotophilus kuhlii*	62			1	
*Cynopterus brachyotis*				1	
*Eonycteris spelaea*			1		
*Macroglossus minimus*	1			2	1
*Ptenochirus jagori*	8				
*Pteropus hypomelanus*			14		
*Rousettus amplexicaudatus*		21	28	1	

Of the 231 bat sera tested, 22 (9.5%) were positive for neutralizing antibodies against ABLV. Antibody titration studies demonstrated decreasing percent neutralization at progressively higher serum dilutions. Of those 22 bat sera, 8 demonstrated no virus neutralization at the next highest dilution tested; 8 demonstrated some neutralization as dilute as 1:40; 3 had some at 1:80; 2 had some at 1:160; and 1 had evidence of some neutralization at a dilution of 1:320. When the strict definition of 90% to be considered positive was used, only two bat sera remained positive at the 1:40 dilution. This dilution is the equivalent of 0.6 IU/mL antibody. Five of those 22 samples were also positive when tested against CVS-11. Only 1 of the 209 bat sera that was negative when tested against ABLV was positive when tested against CVS-11.

The 22 bats with neutralizing antibodies against ABLV included six different species collected from four islands (Table 3). No location was significantly associated with bat sera that tested positive. Antibody-positive bats were evenly dispersed throughout the collection period (July 5 through September 5). Only 32% of the antibody-positive bat sera were obtained from females. That proportion was not statistically significant. The only significant association in the analysis was that a single species had a statistically greater proportion of samples testing positive. Thirty-six percent of the 11 *Mineopterus schreibersi* (Schreiber’s long-fingered bat) tested positive (p=0.01).

**Table 3:** All bats caught on four islands in the Philippines positive for neutralizing antibodies against *Australian bat lyssavirus* at a 1:10 serum dilution

Bat species	Island of origin			
	Luzon	Bohol	Boracay	Mindanao
*Taphozous melanopogan*	4			
*Mineopterus schreibersi*				4
*Philetor brachypterus*				1
*Scotophilus kuhlii*	4			
*Pteropus hypomelanus*			3	
*Rousettus amplexicaudatus*		4	2	

The data analysis was repeated with a less strict case definition of 75% reduction in infectious centers relative to the positive control and including the 43 additional samples that could only be screened at higher dilutions. When these criteria were used, 53 (19%) of 274 bat sera tested were positive. The two samples with the highest positive endpoint titers in the initial analysis remained highest, but now at a 1:80 dilution. Although additional species would have been identified as having neutralizing antibodies, *M. schreibersi* remained the only species with a statistically significantly greater proportion of serum samples positive for neutralizing antibody.

## Discussion

This study presents evidence of neutralization of ABLV by serum from Philippine bats. This neutralizing activity correlated with the ability to neutralize RABV (CVS-11) and titrated steadily with serial dilutions of the serum. These findings are consistent with the presence of naturally occurring antibodies against a *Lyssavirus* related to ABLV in the Philippine bat populations studied.

Lyssaviruses are classified into groups on the basis of their relative pathogenicity, their binding affinity to specific monoclonal antibodies, and their nucleic acid sequences. There are seven putative genotypes that have been aggregated into two basic groups on the basis of their overall phylogenetic relatedness (*19*). Phylogroup I includes RABV (genotype 1), *Duvenhage virus* (DUVV) (genotype 4), *European bat lyssavirus* (EBLV) 1 (genotype 5), EBLV-2 (genotype 6), and ABLV (genotype 7). Phylogroup II includes *Lagos bat virus* (LBV) (genotype 2) and *Mokola virus* (MOKV) (genotype 3). Antibodies to viruses within one phylogroup should cross-neutralize viruses of that same phylogroup. The ability to cross-neutralize is directly proportional to the relative nucleotide and amino acid homogeneity between the two viruses being compared (*19*). In another study of Nigerian fruit bats, 2 of 50 serum samples had neutralizing antibodies against CVS-11 but failed to neutralize DUVV (*20*). In the bats in this study, no cases of active *Lyssavirus* infection were discovered from which nucleotide and amino acid sequences could be determined and subsequently compared with ABLV and CVS-11. More samples neutralized ABLV than CVS-11, suggesting that the *Lyssavirus* responsible for the induction of antibodies in these bats might be more similar to ABLV than CVS-11, while still being a member of phylogroup I. Repeating the RFFIT assays with a challenge virus from phylogroup II, such as MOKV or LBV, could have tested this hypothesis further. Although it is possible that we might have been able to demonstrate some cross-reactivity, a finding of greater neutralization activity against MOKV (compared with what was found against ABLV) would not be expected since all known phylogroup II viruses have a rather limited geographic distribution in Africa. In addition to the more widespread distribution of phylogroup I lyssaviruses, the small quantities of available bat sera precluded repeated RFFIT testing with an additional challenge virus such as MOKV.

The strict case definition used in the interpretation of the RFFIT assays resulted in the identification of 22 positive bat sera. As noted in the results of the second analysis, for which a lower threshold for positivity was used, additional bat sera that had 75% to 89% neutralization also had progressively decreasing neutralization at increasing dilutions, a finding similar to that for the 22 positive samples that met the strict case definition. Thus, we may have slightly underestimated the actual prevalence of anti-*Lyssavirus* antibody in these bat populations. Reduction in infectious centers by 90% compared with the positive control provided a conservative estimate of the prevalence of anti-*Lyssavirus* neutralizing antibodies. Previous studies have used a cutoff as low as 50% neutralization for the interpretation of data (*21*). Although the number of positive bat sera more than doubled when the broader case definition was used, no change in the results of the analysis of patterns of seropositivity by location or type of bat was evident. In addition, independent of the cutoff point used, the peak antibody measurement was approximately 0.6 IU/mL. Most of the other positive specimens had approximately 0.3 IU/L. Many commercial laboratories report serum samples with ≥0.5 IU/mL of antibody as a positive test. However, the 0.5 IU/mL value was established as an arbitrary standard by reference laboratories as evidence above background for the detection of the induction of RABV-neutralizing antibodies in humans after receipt of multiple doses of high-potency rabies vaccines (*22*). No accepted standard for naturally occurring infections among wildlife exists.

On the basis of the 9.5% prevalence of neutralizing antibodies, it is not surprising that all brain samples studied showed no evidence of RABV antigen by DFA in these clinically normal bats. Most studies of healthy bats have found a low prevalence of active infection, usually <1% (*21*). As would be expected, previous surveys of healthy bats in other parts of the world have shown that the prevalence of RABV-neutralizing antibodies is usually considerably higher than the prevalence of active infection, as indicated by positive DFA results for brain tissue. In a study of asymptomatic Mexican free-tailed bats from a single dense cave population in New Mexico, 69% of the bats had neutralizing antibodies, but only 0.5% had active infection demonstrated by DFA (*21*). A report from the Caribbean described a 40.5% seroprevalence of RABV-neutralizing antibodies among healthy and ill bats, but only 1 bat, which had been submitted as an ill-appearing rabies suspect, had active infection (*23*). Assuming a ratio of seroprevalence to active infection of approximately 100:1 in healthy populations of bats, based on the seroprevalence of 9.5% demonstrated in this study, one would have needed to test at least 1,052 normal bats to detect one case of active viral infection by DFA. Such further studies should be focused on species of bats such as *M. schreibersi* and in locations with the highest prevalence of neutralizing antibodies. Ideally, if a large stable colony of such bats could be identified, surveillance among sick and dying bats could be conducted. Such a study would increase the likelihood of obtaining a virus isolate and would minimize the potential adverse impact on the bat populations from oversampling large numbers of otherwise healthy bats. Similarly, the routine virus variant typing of human and domestic animal rabies cases in the Philippines and throughout Southeast Asia will provide basic epidemiologic information on the prevalence of different RABV isolates and enhance the likelihood of discovery of any new lyssaviruses affecting the populations in these regions.
